# Bedside Doppler ultrasound for the assessment of renal perfusion in the ICU: advantages and limitations of the available techniques

**DOI:** 10.1186/s13089-015-0024-6

**Published:** 2015-05-28

**Authors:** David Schnell, Michael Darmon

**Affiliations:** Medical-Surgical Intensive Care Unit, Angoulême Hospital, Angoulême, France; Medical-Surgical Intensive Care Unit, Saint-Etienne University Hospital, Avenue Albert Raimond, 42270 Saint-Priest-en-Jarez, Saint-Etienne France; Jacques Lisfranc Medical School, Jean Monnet University, Saint-Etienne, France

**Keywords:** Acute kidney injury, Resistive index, Doppler, Contrast-enhanced ultrasonography

## Abstract

Three Doppler-derived techniques have been proposed to assess renal perfusion at bedside: Doppler-based renal resistive index (RI) which has been extensively but imperfectly studied in assessing renal allograft status and changes in renal perfusion in critically ill patients and for predicting the reversibility of an acute kidney injury (AKI), semi-quantitative evaluation of renal perfusion using colour-Doppler which may be easier to perform and may give similar information than RI and contrast-enhanced sonography that may allow more precise renal and cortical perfusion assessment. These promising tools have several obvious advantages including their feasibility, non-invasiveness, repeatability and potential interest in assessing renal function or perfusion. However, several limits need to be taken into account with these techniques, and promising results remain associated with large areas of uncertainty. This editorial will describe more carefully advantages and limits of these techniques and will discuss their potential interest in assessing renal perfusion.

**Electronic supplementary material:**

The online version of this article (doi:10.1186/s13089-015-0024-6) contains supplementary material, which is available to authorized users.

Acute kidney injury remains associated with a high morbidity, a high short-term and long-term mortality and a tremendous economic impact [1]. Adequate preventive and curative strategies in this setting are still lacking. Indeed, specific strategies or medications tested over the last decades have been either inefficient or insufficiently validated to be recommended routinely [2]. The usual marker of acute kidney injury (AKI) is either poorly specific, poorly sensitive and/or delayed as regards the renal injury leading to an unavoidable late recognition of AKI and thus delayed interventions that may partly explain this situation [3]. Monitoring renal perfusion may theoretically allow individualized hemodynamic optimization that could ultimately limit renal dysfunction or AKI progression. This hypothesis is currently being investigated in an ongoing study assessing mean arterial level optimization through Doppler-based resistive index (RI) in patients with septic shock (NCT01473498).

Ultrasonography (US) is performed routinely to assess renal and collecting system morphology [4]. B-mode US provides valuable information regarding anatomic features and pathological findings including search for signs suggestive of chronic renal disease, hydronephrosis, calcifications, cysts or solid masses. Renal Doppler US has been used to assess vasculature of either native or transplanted kidneys [5]. Although renal Doppler is valuable for assessing large arterial or venous abnormalities, this technique has been advocated in evaluating changes in intra-renal perfusion due to kidney diseases [6, 7]. Three techniques have been evaluated with various results, interest and limitations in this field: Doppler-based renal RI which has been extensively but imperfectly studied in assessing renal allograft status [8, 9] and changes in renal perfusion in critically ill patients [10–12] and for predicting the reversibility of an AKI [13, 14], semi-quantitative evaluation of renal perfusion using colour-Doppler has been recently suggested and may be easier to perform for similar information than RI [4, 15] and last contrast-enhanced sonography that may be more accurate in assessing renal perfusion [16].

All these techniques are rapid, non-invasive and repeatable, and they may help in assessing renal preclinical dysfunction or vascular damages, in evaluating risk of subsequent renal dysfunction and evaluating the severity of an acute kidney injury. Additionally, both RI and semi-quantitative evaluation of renal perfusion can be performed in most patients by inexperienced operators following a half-day course [15]. On the other hand, reliability seems to be limited, clinical significance uncertain and diagnostic performance remains to be validated in adequately powered studies.

Doppler-based RI is an easy-to-perform evaluation usually obtained from a posterolateral approach [4, 7]. B mode allows location of the kidneys and detection of signs of chronic renal damage. Colour-Doppler or power-Doppler US allows then vessels’ localization and a semi-quantitative evaluation of renal perfusion using colour-Doppler (Table 1 and Additional file 1: Figure S1a) [4]. Either the arcuate arteries or the interlobar arteries are then insonated with pulsed wave Doppler and RI (Pourcelot index) calculated according to the following formula: *RI* = [[*peak systolic shift* − *minimum diastolic shift*]/*peak systolic shift*] (Additional file 1: Figure S1b) [4, 7]. If performed carefully, this measurement is reliable and the side-to-side difference is usually less than 5 % [17]. Renal RI may therefore appear as a simple and non-invasive tool easy to use at the patient bedside. Feasibility of the measure has been showed to be good, even in the settings of critically ill patients, and a recent study suggested a half-day training session might allow inexperienced operators to perform renal Doppler [15]. Interobserver reproducibility of RI measurement by senior radiologist or senior intensivist is considered excellent [13, 18]. In critically ill patients, the interobserver reproducibility between a senior and inexperienced operator is good and measures seem accurate (absence of systematic bias) although associated with a lack of precision (interobserver 95 % confidence interval of ±0.1) [15]. This lack of precision raises questions as regards the ability of RI to assess small changes in renal perfusion or to be valuable in assessing sequential changes related to therapeutic interventions.

**Table 1:** Colour-Doppler for a semi-quantitative evaluation of intra-renal vascularization [4]

Stage	Quality of renal perfusion by colour-Doppler
0	Unidentifiable vessels
1	Few vessels in the vicinity of the hilum
2	Hilar and interlobar vessels in most of the renal parenchyma
3	Renal vessels identifiable until the arcuate arteries in the entire field of view

Bedside Doppler Ultrasound for the assessment of renal perfusion in the ICU: advantages and limitations of the available techniques. David SCHNELL et al.

Doppler-based RI has been proposed to monitor renal perfusion in critically ill patients and to assess impact on renal perfusion of low-dose dopamine infusion and of gradual changes in mean arterial pressure in response to norepinephrine infusion or a fluid challenge [10, 12, 19]. The obtained results, although interesting, are difficult to interpret. First, as suggested by its name, the RI was initially considered an indicator of renal vascular resistance and blood flow. However, experimental and clinical studies have suggested correlation of RI with vascular resistance and blood flow to be weak [20, 21]. The relationship between vascular resistance and RI is therefore limited even with large, non-physiological, pharmacologically induced changes in renal vascular resistances (RI changes of 0.047 IU (±0.008) per logarithmic increase in renal resistances) [22]. Moreover, these changes are to be considered in light of the limited precision of the technique (intra-observer 95 % CI of ±0.05) [13, 14]. In addition, this relationship seems to be linear only when vascular compliance is normal and progressively disappeared when vascular compliance decreases [20–22]. Decreased vascular compliance may result not only from pre-existing subclinical vascular stiffness but also from acute change in renal or intra-abdominal pressure. Thus, the kidney being a capsulated organ, interstitial edema resulting from renal insult translates into increased subcapsular pressure ultimately decreasing renal perfusion [23] and being likely to decrease renal vascular compliance while increasing RI. Similarly, intra-abdominal hypertension, or renal allograft compartment syndrome in renal transplant recipients, has been associated with increased RI [24, 25]. Several additional factors, including heart rate, mean arterial pressure, oxygen and carbon dioxide levels and age also influence RI value [7]. These experimental data, along with results of large studies in renal transplant recipients suggest therefore Doppler-based RI to be an integrative parameter rather than a substitute for renal biopsy or renal perfusion assessment [7, 9]. Despite its limitation, this integrative parameter may help in detecting early renal dysfunction or in predicting short-term reversibility of AKI [6, 13, 14, 26]. Although preliminary results in this field are promising (Fig. 1), discrepant results have been published in this setting [27], and confirmatory studies are still needed.

**Fig. 1:**
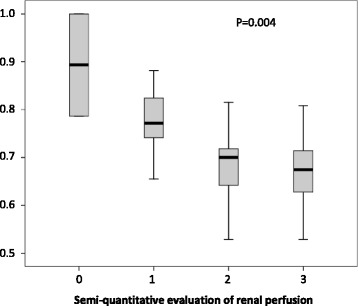
Boxplot of Doppler sonography renal resistive index (RI) values according to semi-quantitative colour-Doppler grade (Reproduced from Schnell et al. Minerva Anesthesiologica 2014 [15])

Semi-quantitative renal perfusion assessment using colour-Doppler seems to be easier to perform than Doppler-based RI and may provide similar information regarding renal function [4, 15]. Renal perfusion is semi-quantitatively assessed using a four-stage scale (Table 1 and Additional file 1: Figure S1a). Results of both RI and semi-quantitative renal perfusion assessment are correlated (Fig. 1), and reliability of the semi-quantitative renal perfusion seems to be similar to those of RI [15]. Similarly, semi-quantitative renal perfusion assessment seems to be associated with short-term reversibility of renal dysfunction which may theoretically help in predicting the need for renal replacement therapy and therefore optimal timing of this technique (Fig. 1) [15]. To the best of our knowledge, however, only a single preliminary report assessed the performance of this technique, and validation of these results are also needed.

Last, contrast-enhanced ultrasonography (CEUS) may be more accurate than the previously described techniques in assessing specifically renal perfusion. This technique associates low mechanical index (MI) ultrasonography and microbubble-based contrast agents and has been proposed to assess organ perfusion [16]. Hence, the intravascular distribution microbubbles along with their sensitivity to high-power acoustic pulses have been used to obtain destruction-refilling sequences, from which some parameters proportional to blood flow can be derived [16]. In a preliminary study performed in healthy volunteers, Schneider and colleagues demonstrated CEUS to detect renal cortical changes following angiotensine II or captopril administration [28]. Additionally, these changes were parallel to those of estimated renal plasma flow as estimated by para-aminohippurate clearance [28]. Two recent studies in critically ill patients assessed changes in CEUS-derived parameters in patients following either terlipressin [29] or norepinephrine administration [30]. Although CEUS was able to found significant changes in cortical perfusion, responses across patients were heterogeneous, unpredictable and of unclear relationship with patients’ characteristics [29, 30]. Last, interobserver variability was suggested to be high (up to 25 %) and discordant changes across perfusion parameters (suggesting in the same patients discordant changes in renal perfusion) found in up to 25 % of the tested patients [30]. Hence, despite a promising initial report, CEUS has still to be demonstrated as both an accurate predictor of renal perfusion and a useful tool for clinical purpose.

These three techniques are promising. Renal Doppler-based resistive index is easy to perform, rapid, non-invasive and repeatable. It is, however, doubtful as regards the numerous factors involved in RI, from the weak relationship between RI and renal vascular resistances to the large interobserver variation, that this tool could accurately assess renal perfusion changes. Nevertheless, renal Doppler has been found to be of potential interest in detecting subclinical renal vascular insults and in evaluating renal prognosis or short-term reversibility of an acute kidney injury and may therefore be clinically relevant at bedside in these indications. Preliminary data regarding semi-quantitative renal perfusion assessment suggest that this technique is easier to perform than RI and may provide similar information. Last, contrast-enhanced ultrasonography may theoretically be more accurate for bedside monitoring of renal perfusion than Doppler-based RI. However, despite promising results in healthy volunteers, this technique still has to be validated in this indication. More importantly, even though these techniques may prove to be useful in assessing renal function or renal perfusion, their clinical inputs at bedside for monitoring, preventive or therapeutic purposes remain theoretical and will have to be assessed.

## Additional file

Additional file 1: Figure S1. Renal colour-Doppler ultrasonography showing renal vascularization and allowing semi-quantitative renal perfusion assessment (S1a). RI measurement using pulsed wave Doppler (S1b). Figures reproduced from Schnell et al. Intensive Care Medicine with authorization [7].

## Abbreviations

AKI: Acute kidney injury
CEUS: Contrast-enhanced ultrasonography
MI: Mechanical index (PNP/√*f*, PNP being the peak negative pressure of the ultrasound wave derated by 0.3 dB cm^−1^ Mhz^−1^ in megapascal and *f* being the centre frequency of the ultrasound wave in MHz)
RI: Doppler-based renal resistive index
US: Ultrasonography
