# Supplementary material for: Bedside Doppler ultrasound for the assessment of renal perfusion in the ICU: advantages and limitations of the available techniques
Source: Crit Ultrasound J. 2015 May 28;7:8. doi: 10.1186/s13089-015-0024-6 (PMC4461647; doi:10.1186/s13089-015-0024-6)

**Figure S1- Renal colour-Doppler ultrasonography showing renal vascularization and allowing semi-quantitative renal perfusion assessment (S1a). RI measurement using pulsed wave Doppler (S1b). Figures reproduced from Schnell et al. Intensive Care Medicine with authorization [7].**

**Figure S1a-**


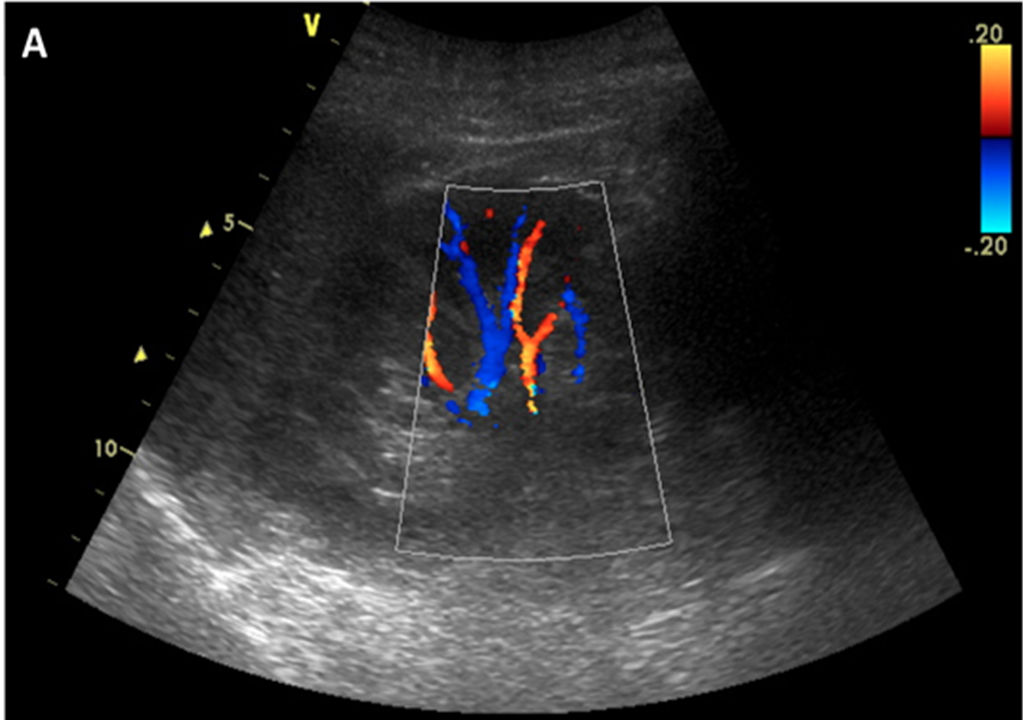


**Figure S1b-**


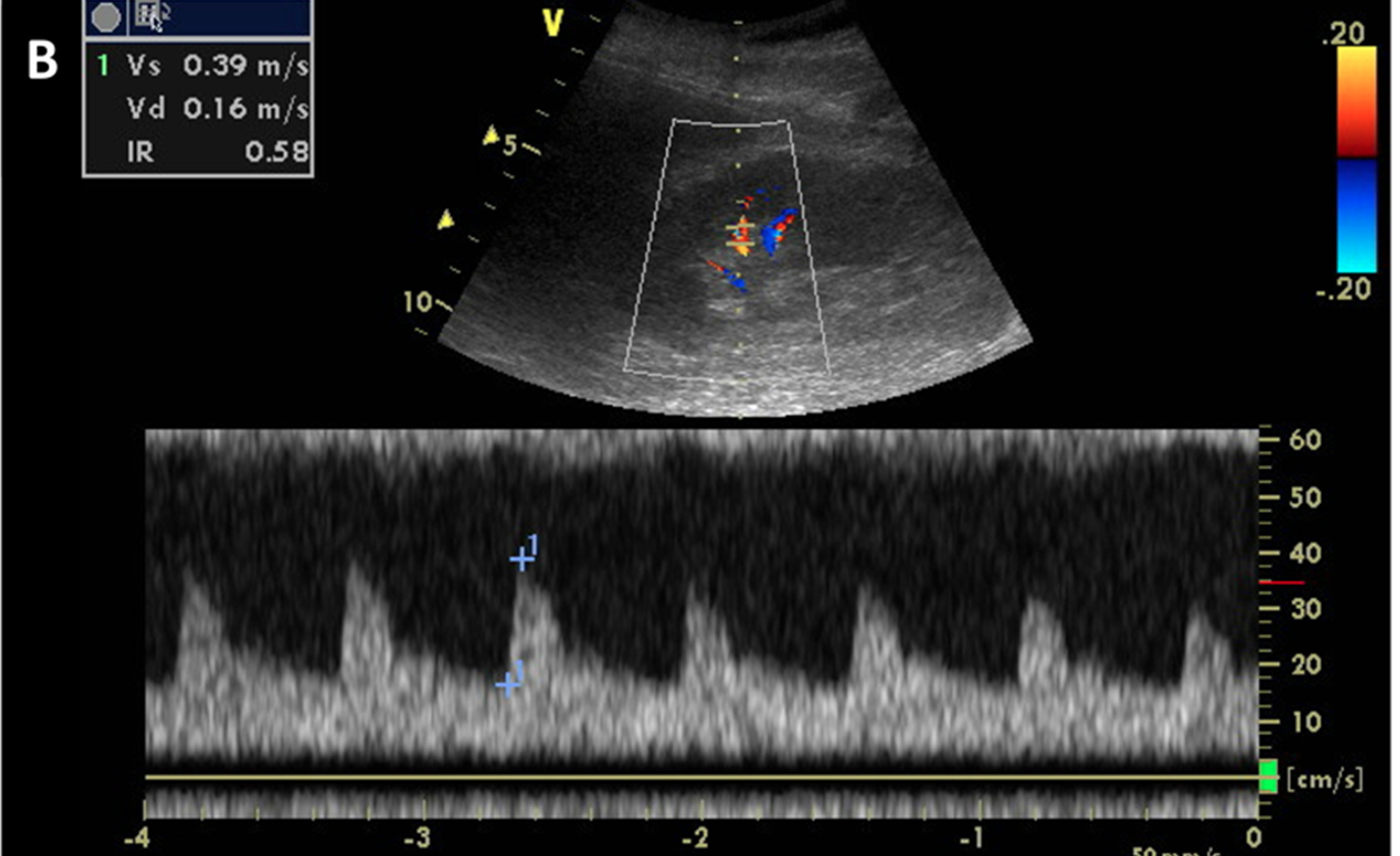

Supplement: Additional file 1: Figure S1. — Renal colour-Doppler ultrasonography showing renal vascularization and allowing semi-quantitative renal perfusion assessment (S1a). RI measurement using pulsed wave Doppler (S1b). Figures reproduced from Schnell et al. Intensive Care Medicine with authorization [7]. [file 13089_2015_24_MOESM1_ESM.doc]
